# Voluntary assignments during the pediatric clerkship to enhance the clinical experiences of medical students in the United States

**DOI:** 10.3352/jeehp.2020.17.17

**Published:** 2020-05-27

**Authors:** Conrad Krawiec, Abigail Kate Myers

**Affiliations:** 1 Division of Pediatric Critical Care Medicine, Department of Pediatrics, Penn State Hershey Children's Hospital, Hershey, PA, USA; 2 General Pediatrics, Department of Pediatrics, Penn State Children's Hospital, Hershey, PA, USA; Hallym University, Korea

**Keywords:** Undergraduate medical education, Educational measurement, Clinical clerkship, Patient-focused care

## Abstract

**Purpose:**

Pediatric clerkships that utilize off-campus clinical sites ensure clinical comparability by requiring completion of patient-focused tasks. Some tasks may not be attainable (especially off-campus); thus, they are not assigned. The objective of this study was to evaluate the feasibility of providing a voluntary assignment list to third-year medical students in their pediatric clerkship.

**Methods:**

This is a retrospective single-center cross-sectional analysis of voluntary assignment completion during the 2019–2020 academic year. Third-year medical students were provided a voluntary assignment list (observe a procedure, use an interpreter phone to obtain a pediatric history, ask a preceptor to critique a clinical note, and follow-up on a patient after the rotation ends). Descriptive statistics were used to assess the timing and distribution of voluntary assignment completion.

**Results:**

In total, 132 subjects (77 on the main campus, 55 off-campus) were included. Eighteen (13.6%) main-campus and 16 (12.1%) off-campus students completed at least 1 voluntary assignment. The following voluntary assignments were completed: observe a procedure (15, 11.4%), use an interpreter phone (26, 19.7%), ask a preceptor to critique a clinical note (12, 9.1%), and follow-up on a patient after the rotation ends (7, 5.3%). Off-campus students completed the assignments more often (29.1%) than on-campus students (23.4%)

**Conclusion:**

Our clerkship values specific patient-focused tasks that may enhance student development, but are not attainable at all clinical sites. When provided a voluntary assignment list, 34 out of 132 students (25.8%) completed them. Clerkships that utilize off-campus sites should consider this approach to optimize the pediatric educational experience.

## Introduction

The aim of the pediatric clerkship is to ensure that students are exposed to various clinical experiences to attain proficiency in multiple core competencies [1,2]. The number of students who can participate in a rotation can be limited if a clerkship primarily utilizes university-based (on-campus) clinical experiences. Thus, medical education departments often offer students patient care opportunities at off-campus clinical sites [3-5]. Off-campus clinical experiences may be community-based, and the type of faculty available and patient population may be different than what students encounter in university-based settings [1,5,6]. According to previous research, however, students who rotate at off-campus sites can meet clerkship objectives, do not have significant differences in performance when compared to on-campus students, and can participate in the patient encounters outlined in the clerkship’s syllabus [4,7]. These clinical settings also can be invaluable for a student’s education as students are exposed to both common and complex problems and may have more opportunities for hands-on experiences [4,7].

The Liaison Committee on Medical Education requires that all clinical sites (on-campus and off-campus) utilized to meet clerkship objectives must be comparable [8]. By ensuring that students have similar clinical experiences and quality of training, barriers to achieving clerkship learning objectives are prevented. Clerkships not only assess and reassess a clinical site each year but also ensure that patient-focused tasks (such as seeing a specific patient type), are available and are attainable at all sites [8]. Because the patient experiences available at clinical sites vary, some students experience the underutilization of assignments that may be considered subjectively important to physician development and allow active participation (such as observing a lumbar puncture) [6]. Potential reasons for this include a lack of patient acuity—and thereby a lack of certain opportunities—at a particular site [9]. This decreased exposure to certain clinical activities can have profound implications for physician development and career selection [10,11]. An alternative approach may be not only to ensure that students participate in clinical activities that meet the clerkship objectives, but also to encourage the completion of voluntary assignments without mandating their completion. Such an approach may highlight an assignment’s relevance and enhance the robustness of the clerkship clinical experience.

This study aimed to evaluate the feasibility of providing a voluntary assignment list to third-year medical students in their pediatric clerkship. We hypothesized that the students would utilize and work to complete a voluntary list of assignments to further enhance their clerkship experience.

## Methods

### Ethics statement

This study was reviewed by the Institutional Review Board at the Penn State College of Medicine in Hershey, PA, USA (STUDY00014593) and determined to be non-clinical research.

### Study design

This was a single-institution cross-sectional descriptive analysis among third-year medical students assigned to complete their required pediatric clerkship at Penn State College of Medicine. A retrospective review of submitted voluntary assignments was completed during the 2019–2020 academic year.

### Subjects

In total, 132 subjects who were part of our school’s traditional curriculum and rotated at the pediatric clerkship’s primary site and at our off-campus affiliate sites were included in this study. Subjects who were part of our integrated longitudinal curriculum were excluded.

### Clerkship overview

The pediatric clerkship is a 4-week rotation with the following clinical requirements: approximately 2 weeks of outpatient care (consisting of well-child checks as well as acute visits), 2 days of the newborn nursery, and 2 weeks of inpatient care on either the pediatric hospitalist service or a pediatric subspecialty service. During the pediatric clerkship, students were required to complete the following assignments: development of learning goals, direct observation of history-taking and a physical examination, direct observation of a developmental assessment, completion of online virtual cases, completion of patient encounter logs, and solicitation of clinical assessment evaluation forms.

### Voluntary assignment completion

Four assignments were developed by the pediatric clerkship director, who was experienced in inpatient medicine, and reviewed by an outpatient pediatrician. They were chosen based on their likelihood of completion according to the authors’ experiences with our medical students, their relevance to clinical practice, and because they subjectively incorporated principles of humanistic care, accountability, and patient ownership.

### Voluntary assignment implementation

Starting on March 4, 2019, the students were advised during the clerkship orientation of the availability of these additional voluntary activities. They were informed that they would not receive credit for these assignments, but were encouraged nevertheless to complete these assignments to enhance their growth and development as physicians. Written instructions on how to complete the assignments were provided via our learning management system. Preceptors were advised of the purpose and voluntary nature of the assignments in written form via e-mail. Pediatric residents were provided a live clerkship curriculum update highlighting the voluntary assignments. The study period ended on the last day of the academic year on December 20, 2019.

### Type and content of voluntary assignments

The 4 voluntary assignments were as follows: observe a procedure (e.g., lumbar puncture), use an interpreter phone to obtain a pediatric history, ask a preceptor to critique a clinical note, and follow-up on a patient after the rotation ends. For the procedure observation assignment, students were asked to describe the indications and contraindications of the procedure, what was observed, and what was learned from the experience. By encouraging students to seek out rare procedural opportunities in pediatrics, we hoped to increase the likelihood that students would observe a different aspect of pediatric care, engage students to participate in these procedures, and enable observation of interprofessional collaboration between physicians and nurses [10]. For the use of an interpreter phone to obtain a pediatric history, students were asked to write a self-reflection note on this clinical experience and whether this activity enriched their clerkship experience (and if not, why not). Language access is a right for individuals with limited English proficiency that can be achieved through medical interpreter services [12]. Nonetheless, it is a skill that requires practice and can be underutilized if not encouraged [12]. This assignment was selected to promote the use of medical interpreter services and to help students develop an empathetic humanistic relationship with patients with limited English proficiency [13]. For asking the preceptor to critique a clinical note, students were asked to upload the original note and describe how they changed the note to improve it. This task was based on learner-driven feedback models, where learners are encouraged to take an active role in the evaluation of their performance to promote student accountability [14]. For follow-up on a patient after the rotation ends, students were asked to write a reflection note on why the patient was admitted to the hospital, why the student chose to follow up on this particular patient, and what the student learned from the experience. We selected this task based on studies in the literature suggesting that continuity of care, even if difficult to achieve between rotations, provides an opportunity for learners to “take ownership” of their patients [15].

### Data collection

All completed assignments were verified using the Canvas (Instructure, Salt Lake City, UT, USA) learning management system. Information was recorded on the timing of the rotation (e.g., the first rotation of the 2019–2020 academic year) and the location where the students completed their clinical experiences (on-campus versus off-campus). Using the Canvas learning management system, we extracted the following data: completion of either observing a procedure (e.g., lumbar puncture), using an interpreter phone to obtain a pediatric history, asking a preceptor to critique a clinical note, or following up on a patient after the rotation ends. We quantified the total amount of students who completed these voluntary assignments for each 4-week rotation and the type of voluntary assignments completed.

### Statistical methods

We used descriptive statistics to assess the study population in terms of timing and the distribution of voluntary assignment submission among the study subjects. The results are presented as frequencies.

## Results

### Overview

Of the subjects, 77 (58.3%) were on the main campus, while 55 subjects (41.7%) completed their clinical experiences off-campus. During the 2019–2020 academic year, 34 subjects (25.8%) completed at least 1 voluntary assignment (Table 1, Dataset 1).

### Type of voluntary assignment completion

The distribution of completion of the voluntary assignments was as follows: observe a procedure, 15 (11.4%), use an interpreter phone, 26 (19.7%); ask a preceptor to critique a clinical note, 12 (9.1%), and follow-up on a patient after the rotation ends, 7 (5.3%) (Table 1).

### On-campus versus off-campus voluntary assignment completion

Eighteen (13.6%) on-campus and 16 (12.1%) off-campus students completed at least 1 voluntary assignment. The on-campus subjects completed voluntary assignments at the following frequencies: observe a procedure, 9 (6.8%); use an interpreter phone, 13 (9.8%); ask a preceptor to critique a clinical note, 9 (6.8%); and follow-up on a patient after the rotation ends, 3 (2.3%). Off-campus subjects’ frequency of completing voluntary assignments was as follows: observe a procedure, 6 (4.5%); use an interpreter phone, 13 (9.8%); ask a preceptor to critique a clinical note, 3 (2.3%), and follow-up on a patient after the rotation ends, 4 (3.0%) (Table 1).

### Timing of voluntary assignment completion

**Fig. 1.**
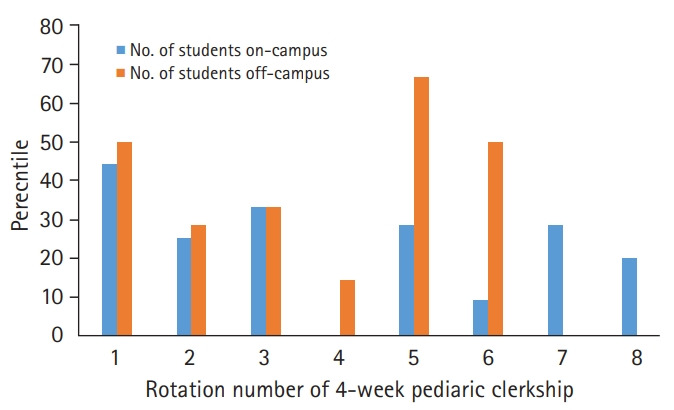
Voluntary assignment completion rate per 4-week pediatric clerkship rotation.

The subjects completed at least 1 voluntary assignment for a majority of the 4-week rotations. For 1 rotation (4), no on-campus subjects completed a voluntary assignment. For 2 rotations (7 and 8), no off-campus subjects completed a voluntary assignment. The subjects completed voluntary assignments at a greater frequency for the first 6 rotations of the academic year. The last 3 rotations had a lower frequency of voluntary assignment completion (Fig. 1, Supplement 1).

## Discussion

We tried to determine whether a large proportion of students would complete at least 1 assignment that could be accomplished at on-campus and off-campus sites by providing a list of voluntary assignments designed to promote students’ growth and development. This study successfully introduced additional educational activities during the clerkship. We demonstrated that on-campus and off-campus students completed at least 1 voluntary assignment, but overall, only 25.8% of students participated. These results imply that roughly a quarter of students will complete voluntary assignments, potentially enhancing their clinical experiences.

There were 3 important, unexpected observations regarding the completion of voluntary assignments. First, a majority of students elected to utilize the interpreter phone to complete their voluntary assignment. This finding demonstrates the ease of accessing this technology within our institution (and within community-based settings), and the frequency with which this assignment was completed may be high enough to consider making it a required assignment. These results also may imply that students understand the value of this particular voluntary assignment. The utilization of a medical interpreter is a patient’s right and should be part of routine practice when caring for patients with limited English-language proficiency. It provides an avenue to acquire essential information on a patient’s history, and it establishes an empathetic relationship with the patient [13]. The frequency of students reporting completion of a voluntary task that is considered a part of the routine clinical workflow suggests that their experience may have been vital to their development as physicians. Further research on this is needed.

Following up on a patient after the rotation ends was the least utilized voluntary assignment, which is not unexpected, as specific learners may experience barriers to achieving continuity of care as they transition to other rotations [15]. However, it should be noted that off-campus students completed this assignment more often (29.1%) than on-campus students (23.4%). It is unknown why this phenomenon occurred. One possibility is that at the end of the rotation, off-campus students remained at the same site to start their next rotation and had greater ease of access. In a community-based hospital setting, the institution is usually smaller and possibly more close-knit; thus, students had more opportunities for continuity of care. Otherwise, students may have had more opportunities for patient ownership as the number of residents present or the patient census may have been lower in community-based settings than in university-based settings.

Our final observation is that when we assess comparability across clinical sites, the clinical aspects of medical training appear to receive the most attention (i.e., patient type). In the United States, there is now an increased emphasis on physicians attaining humanistic qualities as they undergo their training, in addition to being experienced clinically [13]. While most medical schools ensure that this humanistic aspect is part of the curriculum and can be accomplished on-campus, it is unknown whether it is achievable or whether it is ensured in an off-campus setting. We demonstrated a possible way to assess this issue through assignments focused on humanistic aspects of medicine. Our clerkship will reconsider our approach to ensuring comparability by reviewing the type of assignments we require, selecting assignments that highlight the humanistic aspects of medicine, and ensuring that these aspects are achieved at all sites.

The major limitation was that this was a single-center retrospective study. Because the assignments were student-directed, it is possible that students falsified completion of these assignments. We feel, however, that this is highly unlikely, as these assignments were voluntary and were not considered as part of students’ summative performance. It is unknown whether the low completion rate signals students’ disinterest or indicates that these experiences were simply not available. Thus, in the next academic year, we will continue to provide voluntary assignments. If the completion rate continues to be low, we will administer surveys and convene focus groups to determine the reasons why.

In conclusion, our pediatric clerkship values certain assignments that enhance students’ development, but struggles to ensure that they are attainable at all our clinical sites. Our study demonstrated that when provided a voluntary assignment list, some students utilized it, potentially enhancing their clinical experiences. Clerkships that utilize off-campus sites should consider this approach to optimize the pediatric educational experience.

## Figures and Tables

**Table 1.** Overview of voluntary assignment completion

Variable	On-campus	Off-campus
Total no. of students	77 (58.3)	55 (41.7)
No. of students completing at least 1 assignment	18 (13.6)	16 (12.1)
Type of voluntary assignment completion		
Observe a procedure	9 (6.8)	6 (4.5)
Use an interpreter phone	13 (9.8)	13 (9.8)
Ask a preceptor to critique clinical note	9 (6.8)	3 (2.3)
Follow-up on a patient after the rotation ends	3 (2.3)	4 (3.0)

Values are presented as number (%).

## Footnotes

**Data availability** Data files are available from Harvard Dataverse: https://doi.org/10.7910/DVN/RC6JL4 Dataset 1. Raw data for the medical students’ voluntary assignments during the pediatric clerkship at Penn State College of Medicine.

## Supplementary materials

Data files are available from Harvard Dataverse: https://doi.org/10.7910/DVN/RC6JL4

Supplement 1. Voluntary assignment completion rate of on-campus and off-campus students per 4-week pediatric clerkship rotation.

Supplement 2. Audio recording of the abstract.
