# Supplementary material for: Voluntary assignments during the pediatric clerkship to enhance the clinical experiences of medical students in the United States
Source: J Educ Eval Health Prof. 2020 May 27;17:17. doi: 10.3352/jeehp.2020.17.17 (PMC7338279; doi:10.3352/jeehp.2020.17.17)
Supplement: Supplementary file 2 — Supplement 1. Voluntary assignment completion rate of on-campus and off-campus students per 4-week pediatric clerkship rotation. [file jeehp-17-17-suppl.pdf]

Suppl. 1. Voluntary assignment completion rate of students of on-campus and off-campus per 4-week pediatric clerkship rotation.

| | On-Campus | | Off-Campus | |
|---|---|---|---|---|
| Rotation | Number of On-Campus Subjects (n) | Assignment Completion (n,%) | Number of Off-Campus Subjects (n) | Assignment Completion (n,%) |
| 1 | 9 | 4 (44.4%) | 6 | 3 (50.0%) |
| 2 | 8 | 2 (25.0%) | 7 | 2 (28.6%) |
| 3 | 9 | 3 (33.3%) | 6 | 2 (33.3%) |
| 4 | 9 | 0 (0.0%) | 7 | 1 (14.3%) |
| 5 | 7 | 2 (28.6%) | 6 | 4 (66.7%) |
| 6 | 11 | 1 (9.1%) | 6 | 3 (50.0%) |
| 7 | 7 | 2 (28.6%) | 5 | 0 (0.0%) |
| 8 | 10 | 2 (20.0%) | 6 | 0 (0.0%) |
